# A sudden change of heart: Warm acclimation can produce a rapid adjustment of maximum heart rate and cardiac thermal sensitivity in rainbow trout

**DOI:** 10.1016/j.crphys.2022.03.003

**Published:** 2022-03-17

**Authors:** Matthew J.H. Gilbert, Olivia A. Adams, Anthony P. Farrell

**Affiliations:** a Department of Zoology, University of British Columbia, #4200 - 6270, University Blvd, Vancouver, BC, V6T 1Z4, Canada; b Faculty of Land and Food Systems, University of British Columbia, 2357 Main Mall, Vancouver, BC, V6T 1Z4, Canada

**Keywords:** Thermal acclimation, Acclimation rate, Fish thermal tolerance, Heart rate, Cardiac function

## Abstract

Warm acclimation in fish is often characterized by an increase in heat tolerance and a reduction in physiological rates to improve the scope to respond to additional challenges including further warming. The speed of these responses can determine their effectiveness. However, acclimation rates vary across levels of biological organization and are poorly understood in part because most research is conducted after an acclimation period of >3 weeks, when acclimation is presumed to be complete. Here we show that when rainbow trout were transferred from 10 to 18 °C, over 50% of the total reduction of maximum heart rate (ƒ_Hmax_) (i.e. the thermal compensation at moderate temperatures) occurred within 72 h, with further compensation occurring more gradually over the following 25 days. Also, the ability to increase ƒ_Hmax_ with acute warming improved within 24 h resulting in a 30% rise in peak ƒ_Hmax_, but this ultimately declined again with prolonged (28 days) exposure to 18 °C. In contrast with some previous studies, upper critical temperatures for ƒ_Hmax_ did not increase. Nonetheless, we demonstrate that rapid cardiac plasticity is possible in rainbow trout and likely blunts the impacts of thermal variation over relatively short timescales, such as that associated with heat waves and migration between water bodies.

## Highlights

• Warm acclimation often increases cardiac heat tolerance and lowers heart rates.
• Rainbow trout were transferred from 10 to 18 °C to assess cardiac acclimation rates.
• Within 24h the ability to increase maximum heart rate with warming increased by ∼30%.
• ∼50% of the reduction of max heart rate at moderate temperatures occurred by 72h.
• Rapid cardiac plasticity likely helps fish mitigate acute thermal challenges.

## 1 Introduction

Fish can compensate for prolonged temperature changes by adjusting their molecular and physiological systems to improve whole-organism performance under the prevailing temperatures. During this thermal acclimation, thermal tolerances are typically shifted in the direction of the temperature change, while physiological rates shift in the opposite direction to help compensate for the immediate thermodynamic effects of the temperature change. Knowing the time course for this thermal acclimation (i.e. acclimation rate) is crucial to understand how fishes mitigate thermal challenges on time scales from minutes to months.

While thermal acclimation likely occurs well within the ecologically relevant timeframe for gradual, prolonged changes in water temperature (such as those between seasons), the rate of acclimation may become limiting for more acute and shorter changes. For example, adult salmonids returning from cooler marine temperatures to much warmer rivers during their physically demanding spawning migration (Eliason et al., 2011; Farrell, 2009; Gilbert and Tierney, 2018) would likely benefit greatly from a rapid thermal compensation of their cardiorespiratory performance.

Yet, our knowledge of the dynamics of thermal acclimation is limited, in part, because most physiological acclimation studies allow for a prolonged acclimation period (commonly 3–4 weeks) and assume that fish have achieved a stable, fully acclimated phenotype (Ekström et al., 2016; Sandblom et al., 2014; Somero, 2010; Sutcliffe et al., 2020). Nevertheless, pioneering studies demonstrated a rapid increase in whole-organism heat-tolerance (e.g. half times of 24–96 h) during warm acclimation (Brett, 1946), something now shown multiple times in temperate and tropical fishes (Bennett et al., 1998; Chung, 1981, 2000, 2001; Fangue et al., 2014). For example, Fangue et al. (2014) found that sheepshead minnows (*Cyprinodon variegatus*) transferred from 11 to 18 °C ultimately increased their acute upper heat tolerance (i.e. critical thermal maximum; CT_max_) by 6.3 °C, but ∼50% of this change occurred within the first 48–72 h. Importantly, this study also found that the acclimation rate for upper and lower critical temperatures varied with the absolute temperature and any gain in thermal tolerance became slower and smaller as a fish approached its limits for plasticity. Such thorough characterizations of thermal acclimation dynamics are rare and the dynamics of thermal acclimation at sub-organismal levels are even less well resolved.

The time course for cardiac thermal acclimation is one particular area in need of further investigation. The heart is a fundamental life support system and warming drives a largely passive increase in cardiac output, which supports elevated tissue oxygen demands (Eliason and Anttila, 2017; Farrell, 2009). During warm acclimation cardiac heat tolerance commonly increases while resting, intrinsic and maximum heart rates (ƒ_Hrest_, ƒ_Hint._, ƒ_Hmax_ respectively) can all decrease (Eliason and Anttila, 2017) to counteract the positive chronotropic effect of warming (i.e. thermal compensation). Components of this response have been identified in polar, temperate and tropical fishes; however, little is known about the time course for these changes (Aho and Vornanen, 2001; Anttila et al., 2014; Drost et al., 2016; Ekström et al., 2016; Ferreira et al., 2014; Gilbert and Farrell, 2021; Safi et al., 2019; Sutcliffe et al., 2020).

To help fill this knowledge gap, we characterized the time course for thermal acclimation of ƒ_Hmax_, its acute thermal sensitivity (i.e. incremental temperature coefficient; Q_10_), and cardiac heat tolerance. We focused on ƒ_Hmax_ because, regardless of temperature, ƒ_Hmax_ contributes to setting the upper limits for cardiac output and thus convective oxygen delivery. Furthermore, because routine ƒ_H_ (ƒ_Hroutine_) increases with temperature, ƒ_Hmax_ must also increase with acute warming to maintain scope for ƒ_H_ above ƒ_Hroutine_. However, acute warming typically only increases ƒ_Hmax_ up to a peak or plateau before cardiac performance collapses as the heartbeat loses its rhythmicity (Casselman et al., 2012; Vornanen, 2016; Vornanen et al., 2014), resulting in a loss of scope for ƒ_H_ at high temperatures (Eliason and Anttila, 2017; Eliason et al., 2013). We characterized the acute ƒ_Hmax_ response in anaesthetized rainbow trout (*Oncorhynchus mykiss*) during thermal acclimation from 10 to 18 °C over 28 days at eight different time points starting at 24 h post-transfer. We hypothesized that rainbow trout cardiac thermal performance would initially adjust rapidly (days) during warm acclimation followed by a slower period (weeks) of acclimation. Specifically, we predicted that cardiac heat tolerance would increase and ƒ_Hmax_ would decrease, first rapidly and then more slowly over time.

## 2 Methods

Rainbow trout (Blackwater Strain, Freshwater Fisheries Society of BC, Abbotsford, B.C.) were housed in 500 L cylindrical tanks at 10 °C for at least 4 weeks prior to being acutely transferred to a similar housing system at 18 °C. Partial freshwater recirculation was used at both temperatures to maintain water quality while allowing temperature control. Cardiac assessments were made simultaneously on six fish both prior to transfer (Day 0) and following transfer (1, 2, 3, 5, 7, 14 and 28 days) from 10 to 18 °C. To remove the potential effects of assessment order, the initial transfer dates were staggered so that fish with different acclimation durations were sampled simultaneously.

The cardiac heat tolerance and the response of ƒ_Hmax_ to acute warming were assessed as previously described (Anttila et al., 2014; Casselman et al., 2012; Gilbert and Farrell, 2021). Briefly, fish were anaesthetized (150 mg L^−1^ tricaine methane sulfonate (TMS) buffered 1.5:1 with NaHCO3) at their acclimation temperature and then transferred to the experimental bath at 10 °C with a maintenance concentration of anaesthetic solution (75 mg L^−1^ TMS buffered 1.5:1 with NaHCO_3_) continuously pumped over their gills. After fitting subdermal electrocardiogram (ECG) electrodes (Gilbert, 2020) fish were injected with atropine (1.2 mg kg^−1^) and isoproterenol (4 μg kg^−1^) with a total injection volume of 1 ml kg^−1^ to induce ƒ_Hmax_. All reagents were acquired from Sigma-Aldrich (St. Louis, USA). The acquisition of the ECG signal and the measurement of ƒ_Hmax_ at each temperature increment during the acute warming protocol were as previously described (Gilbert, 2020; Gilbert and Farrell, 2021).

The acute warming protocol started once the ƒ_H_ had stabilized (20–30 min) and consisted of 1 °C increments every 6 min (10 °C h^−1^). The ƒ_Hmax_ was recorded over the final minute at each temperature increment. The experiment ended once the heartbeat became arrhythmic, at which point fish were euthanized with an overdose of anaesthetic followed by pithing. The onset of arrhythmia was always abrupt and clearly apparent as entirely skipped heart beats which resulted in a sudden, at least, halving of instantaneous heart rate. Body mass, fork length and relative ventricular mass (RVM; Ventricle mass*body mass^−1^ *100) were measured following euthanization.

The peak ƒ_Hmax_, the temperature at which it occurred (T_peak_) and the temperature for the first onset of cardiac arrhythmia (T_arr_) were recorded as indicators of cardiac performance. Also, the incremental Q_10_ temperature coefficient for ƒ_Hmax_ (Anttila et al., 2013; Gilbert, 2020) was calculated over every 2 °C temperature increment and the temperature at which it fell below 2.0 (T_Q10<2.0_) was used as another cardiac transition temperature. The capacity to increase ƒ_Hmax_ during acute warming above each acclimation temperature was calculated as the difference between the peak ƒ_Hmax_ and the ƒ_Hmax_ recorded at either 10 or 18 °C (Δƒ_H>10_ and Δƒ_H>18_).

The strengths and limitations (e.g. absence of normal autonomic control) of assessing the response of ƒ_Hmax_ to acute warming in anaesthetized fish have been discussed previously (Anttila et al., 2013; Casselman et al., 2012; Gilbert and Farrell, 2021; Gilbert et al., 2020). Briefly, this method is high throughout, improves animal welfare over alternative options, eliminates the confounding influences of behaviour and autonomic control on measurements of ƒ_Hmax_, and produces ƒ_Hmax_ values comparable to those in non-anaesthetized fish with similar peak ƒ_H_ values in response to warming as ƒ_Hroutine_ and ƒ_Hmax_ converge at high temperatures (Adams et al., 2022; Casselman et al., 2012; Ekström et al., 2016; Eliason et al., 2013; Gilbert, 2020; Gilbert et al., 2020; Penney et al., 2014).

All data analysis used R Studio (R Core Team, 2014) and data presentation used Prism v.9 (GraphPad Software, San Diego, USA). Statistical significance was set at p < 0.05. Differences in RVM, ƒ_Hmax_ at 10, 14 and 18 °C, Δƒ_H>10_ and Δƒ_H>18_ among acclimation timepoints were assessed though analysis of variance (ANOVA) and homogeneity of variance and normality of residuals were verified using the Levene's and Shapiro-Wilk tests respectively. Given the large number of pairwise comparisons, pairwise t-tests among all acclimation timepoints were done using the Benjamini–Hochberg procedure to control the false discovery rate. The ƒ_Hmax_ and Q_10_ during acute warming were also assessed using linear mixed effects models (LMM) with ƒ_Hmax_ or Q_10_ as a function of acute temperature and acclimation duration with fish ID included as a random effect to account for repeated measures for individuals (LME4 Package; Bates et al., 2007). Marginal (fixed effects only) and conditional (fixed and random effects) coefficients of determination (R^2^) were calculated for the LMMs using the MuMIn: Multi-Model Inference package (Barton, 2015).

## 3 Results

**Fig. 1:**
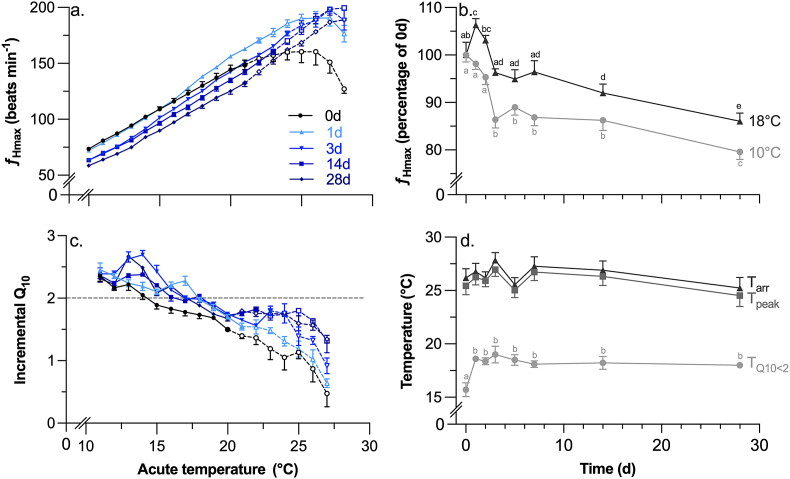
The response of maximum heart rate (ƒ_Hmax_) to acute warming in rainbow trout before (0d) and for 28d following transfer from 10 to 18 °C. Trout were acutely warmed from an initial temperature of 10 °C (a) until their heartbeat became arrhythmic. The ƒ_Hmax_ at 10 and 18 °C over the acclimation period are shown relative to that at 0d (b), along with the incremental Q_10_ (c), and the temperatures at which the Q_10_ fell below 2.0 (T_Q10<2_), the peak ƒ_Hmax_ occurred (T_peak_), and the heartbeat became arrhythmic (T_arr_). Dissimilar letters in panels b. and d. indicate significant differences between timepoints. Dashed lines (a. and c.) indicate temperatures at which individuals were removed after exhibiting arrhythmias.

As expected, ƒ_Hmax_ increased during acute warming in all individuals before the heartbeat became arrhythmic at temperatures individually ranging from 22 to 30 °C (Fig. 1a; Table 1). Likewise, warm acclimation following transfer from 10 to 18 °C reset ƒ_Hmax_ to a lower rate over temperatures between 10 and 20 °C (Fig. 1a and b; Table 1, Table 2). Importantly, no resetting of ƒ_Hmax_ had occurred within the first 48 h of acclimation. However, ƒ_Hmax_ was reduced by 8% after 72 h and by 17% after 28 days (Table 1).

**Table 1:** Morphometrics, maximum heart rate (ƒ_Hmax_), and cardiac thermal performance of rainbow trout sampled before (0d), and at multiple time points after being transferred from 10 to 18 °C water. Data include relative ventricular mass (RVM; percentage of body mass), maximum heart rates (ƒ_Hmax_) at multiple temperatures during acute warming from 10 °C, total increase in ƒ_Hmax_ above 10 and 18 °C during acute warming (Δƒ_Hmax_), and the temperatures at which the incremental Q_10_ for ƒ_Hmax_ fell below 2.0 (T_Q10<2_), ƒ_Hmax_ reached its peak (T_peak_) and the heartbeat became arrhythmic (T_arr_). Dissimilar letters indicate significant differences between timepoints.

		Time (days)							
		0	1	2	3	5	7	14	28
	n	10	10	8	7	8	10	9	9
	length (mm)	91 ± 4	88 ± 3	87 ± 3	90 ± 3	82 ± 5	87 ± 2	78 ± 1	90 ± 3
	mass (g)	8.6 ± 1.5	6.8 ± 0.8	6.7 ± 0.8	6.8 ± 0.7	5.5 ± 0.9	6.5 ± 0.5	4.4 ± 0.2	7.0 ± 0.6
	RVM (%)	0.08 ± 0.005	0.1 ± 0.003	0.08 ± 0.007	0.09 ± 0.008	0.09 ± 0.003	0.09 ± 0.002	0.1 ± 0.006	0.09 ± 0.002
***ƒ***_**H**_**(beats min**^**−1**^**)**	*ƒ*_Hmax_ 10 °C	73.5 ± 1.1^a^	72.2 ± 0.9^a^	70.1 ± 1.1^a^	63.5 ± 1.3^b^	65.5 ± 1.2^b^	63.9 ± 1.3^b^	63.4 ± 1.6^b^	58.5 ± 1.2^c^
	*ƒ*_Hmax_ 14 °C	102.1 ± 1.7^a^	101.2 ± 1.2^a^	99.8 ± 1.3^a^	91.5 ± 1.2^b^	92 ± 1.8^b^	91.6 ± 1.9^b^	89.4 ± 1.8^b^	84.1 ± 1.6^c^
	*ƒ*_Hmax_ 18 °C	130.1 ± 3.5^ab^	138.3 ± 1.7^c^	134.1 ± 1.4^bc^	125.3±1^ad^	123.6 ± 2.5^ad^	125.5 ± 3.1^ad^	119.7 ± 2.4^d^	112 ± 2.2^e^
	peak *ƒ*_Hmax_	163.8 ± 5.6^a^	188.1 ± 5.4^b^	189.1 ± 5.7^b^	191.4 ± 5.1^b^	173.5 ± 4.1^a^	189.3±8^b^	187.7 ± 8.7^b^	159.3 ± 9.1^a^
	Δ *ƒ*_Hmax_ >10 °C	90.2 ± 5.4^a^	115.9 ± 5.4^bc^	119 ± 5.3^bc^	127.8 ± 5.5^b^	108 ± 3.5^ab^	125.4 ± 7.2^b^	124.3 ± 8.1^b^	100.8 ± 8.3^ac^
	Δ *ƒ*_Hmax_ >18 °C	33.7 ± 3.1^a^	49.8 ± 5.1^ab^	55 ± 4.8^ab^	66.1 ± 5.3^b^	49.9 ± 3.2^ab^	63.8 ± 6.5^b^	68 ± 7.7^b^	47.3 ± 7.5^ab^
**T (°C)**	T_Q10<2_	15.7 ± 0.7^a^	18.6 ± 0.3^b^	18.4 ± 0.3^b^	19 ± 0.8^b^	18.5 ± 0.5^b^	18.1 ± 0.3^b^	18.2 ± 0.6^b^	18 ± 0.3^b^
	T_peak_	25.4 ± 0.8	26.2 ± 0.7	25.9 ± 0.6	27 ± 0.6	25 ± 0.7	26.7 ± 0.8	26.3 ± 0.8	24.5 ± 1
	T_arr_	26.2 ± 0.8	26.8 ± 0.8	26.2 ± 0.6	27.8 ± 0.7	25.5 ± 0.7	27.3 ± 0.9	26.9 ± 0.9	25.2 ± 1

**Table 2:** Linear mixed effects model statistics for rainbow trout maximum heart rate (ƒ_Hmax_) and the incremental Q_10_ for ƒ_Hmax_ as a function of the acute exposure temperature (10–20 °C), the acclimation duration (days), and their interaction. Fish ID was included as a random factor to account for repeated measures on an individual. Acute exposure temperatures in the models were restricted to 10–20 °C to exclude high temperatures that constrain ƒ_Hmax_. The interaction term in the incremental Q_10_ model was not statistically significant (F_7,622_ = 1.4, p = 0.201) and was excluded.

		Df	F	p
**ln(**ƒ_Hmax_)				
Acute temp. °C	1692		68505.5	<0.001
Duration	7136		29.5	<0.001
Interaction	7692		35.4	<0.001
	marginal R^2^ = 0.95, conditional R^2^ = 0.99			
**Q**_**10**_**for** ƒ_Hmax_				
Acute temp.	1629		877.3	<0.001
Duration	7,62		11.6	<0.001
	marginal R^2^ = 0.58, conditional R^2^ = 0.60			

The change in the incremental Q_10_ between 10 and 20 °C showed a different, more rapid acclimation rate; it had increased by 11% after 24 h and then remained stable thereafter (Fig. 1c; Table 2). Consequently, T_Q10<2.0_ also increased within 24 h (+18%; Table 1, Table 3) and then remained stable. As a result of this elevated acute thermal sensitivity, peak ƒ_Hmax_ was significantly elevated within 24 h and was up to 17% higher at multiple time points during the first 14 days of acclimation, after which it declined (Table 1, Table 3). Together, this increase in peak ƒ_Hmax_ and the resetting of ƒ_Hmax_ meant that the total Δƒ_Hmax_ above 10 and 18 °C was significantly elevated at multiple points throughout the acclimation period (Table 1, Table 3). Contrary to our expectations, neither T_peak_ nor T_arr_ changed significantly during warm acclimation (Table 1, Table 3).

**Table 3:** Analysis of variance (ANOVA) statistics for the effect of thermal acclimation duration on maximum heart rate (ƒ_Hmax_) metrics and cardiac heat tolerance following acute transfer from 10 to 18 °C. Response variables include ƒ_Hmax_ at multiple temperatures, peak maximum heart rate (peak ƒ_Hmax_), the total increase in ƒ_Hmax_ above 10 and 18 °C during acute warming (Δƒ_Hmax_), and the temperatures at which the incremental Q_10_ for ƒ_Hmax_ fell below 2.0 (T_Q10<2_), ƒ_Hmax_ reached its peak (T_peak_) and the heartbeat became arrhythmic (T_arr_).

		Df	F	p
*ƒ*_H_ (beats min^−1^)	*ƒ*_Hmax_ 10 °C	7,63	18.9	<0.001
	*ƒ*_Hmax_ 14 °C	7,63	16.2	<0.001
	*ƒ*_Hmax_ 18 °C	7,63	11.3	<0.001
	peak *ƒ*_Hmax_	7,63	3.6	0.003
	Δ*ƒ*_Hmax_ >10 °C	7,63	4.4	<0.001
	Δ*ƒ*_Hmax_ >18 °C	7,63	4.2	<0.001
T (°C)	T_Q10<2_	7,63	4.6	<0.001
	T_peak_	7,63	1.1	0.386
	T_arr_	7,63	1.1	0.406

## 4 Discussion

In the present study, we provide the first investigation of the dynamics of thermal acclimation for ƒ_Hmax_ in fish. We show that the majority of the thermal compensation of ƒ_Hmax_ occurred within 72 h, with the resetting of peak ƒ_Hmax_ taking just 24 h and the resetting of ƒ_Hmax_ at a given temperature being detectable between 48 h and 72 h. This rapid acclimation likely has ecological consequences because fishes can experience thermal variation over time scales from minutes to months as they navigate life in spatially and temporally heterogenous thermal environments. For example, farmed, stocked and wild salmonids can all encounter dramatic shifts in temperature as they migrate or are transferred between waterbodies (e.g. ocean to river to lake) (Farrell, 2009; Farrell et al., 2008). Furthermore, diurnal fluctuations (e.g. >10 °C day-1; Gilbert and Tierney, 2018; Rodnick et al., 2004) and weather-related changes such as heatwaves (Wade et al., 2019) are likely to continue increasing in intensity and duration (Woolway et al., 2021).

### 4.1 *Thermal compensation of* ƒ_Hmax_

Routine ƒ_H_, intrinsic ƒ_H_ and ƒ_Hmax_ are commonly reset to lower levels during warm acclimation to counteract the initial cardiac acceleration due to the thermodynamic effect of acute warming, which is an acclimation response also seen for aerobic metabolic rate. For instance, in Arctic char, ƒ_Hmax_ at 10 °C was 16% lower after ∼6 weeks of acclimation to 14 °C compared with acclimation to 6 °C (Gilbert and Farrell, 2021). Similarly, in Atlantic salmon, ƒ_Hmax_ at 12 °C was 12% lower after 3 months of acclimation to 20 °C compared with acclimation to 12 °C (Anttila et al., 2014). For rainbow trout in the present study, ƒ_Hmax_ at 14 °C was reduced by 18% after full (4 weeks) acclimation from 10 to 18 °C (Table 1), but approximately half of this reduction occurred after 48–72 h. This resetting of ƒ_Hmax_ has two likely benefits. First, it ensures that ƒ_Hmax_ is physiologically sustainable and efficient while allowing adequate filling time to maintain stroke volume. Second, it likely allows for an improved capacity to increase ƒ_Hmax_ in response to acute warming (Table 1) when combined with an increased stability of cardiac function at high temperatures as discussed below.

The thermal compensation of ƒ_Hmax_ seen here likely involved, in part, a resetting of the intrinsic cardiac pacemaker rate to a lower level with warm acclimation as this intrinsic rate in rainbow trout can reset in <24 h during warm acclimation (Sutcliffe et al., 2020). Changes in cardiac β-adrenergic receptor density and sensitivity may have also contributed to the observed changes in ƒ_Hmax_ (Keen et al., 1993; Wood et al., 1979). However, these receptors were maximally stimulated in the present study and so we do not know the relative contribution of such changes, nor the relative importance of this important autonomic control of f_H_ *in vivo*.

### 4.2 *Acute thermal sensitivity of* ƒ_Hmax_

Increasing ƒ_H_ during acute warming is the central cardiac response used by fishes to meet elevations in metabolic oxygen demand (Eliason and Anttila, 2017; Farrell, 2009), meaning that if ƒ_Hmax_ does not increase, scope for ƒ_H_ would collapse. To mitigate such constraints, warm acclimation can increase the Δƒ_Hmax_ attainable above the acclimation temperature during acute warming by increasing peak ƒ_Hmax_ (Anttila et al., 2014; Ferreira et al., 2014; Gilbert and Farrell, 2021; Safi et al., 2019). Our results indicate that such a change can occur remarkably quickly. Here, the initial T_Q10<2.0_ for ƒ_Hmax_ (15.7 °C) at 0 d, which was similar to that of another rainbow trout strain acclimated to 10 °C (15–16 °C) (Anttila et al., 2013), increased by 2.9 °C within 24 h of transfer to 18 °C. This increase in T_Q10<2.0_, indicates that ƒ_Hmax_ could continue increasing to a greater extent at higher temperatures during acute warming to support elevated oxygen demand, as reflected by Δƒ_Hmax_>10, Δƒ_Hmax_>18 °C, and peak ƒ_Hmax_ significantly increasing within 24 h of transfer from 10 to 18 °C. Interestingly, these benefits did not persist after 14 d through to 28 d following the transfer. One possible explanation for this decline is that our warm acclimation temperature (18 °C) may have approached the prolonged upper thermal limit for this strain of rainbow trout. Indeed, 18 °C is currently listed as the US Environmental Protection Agency's (US EPA, 2003) regulatory target for the seven-day average daily maximum for rainbow trout habitat. Likewise, in Arctic char, peak ƒ_Hmax_ during acute warming, progressively increased with acclimation temperature from 2 to 14 °C following prolonged acclimation (>6 weeks), but not from 14 to 18 °C (Gilbert and Farrell, 2021), supporting the possibility that too warm an acclimation temperature can impair upper cardiac thermal performance. With multiple rainbow trout strains from a more southerly provenience performing well at a temperature higher than 18 °C (Adams et al., 2022; Chen et al., 2015; Rodnick et al., 2004; Verhille et al., 2016), examinations of their cardiac-thermal acclimation dynamics are warranted. The importance of such transient cardiac acclimation responses has been previously acknowledged (Ekström et al., 2016); By examining only a final acclimation time point (e.g., 28 days is a common acclimation period), aspects of the warm-acclimation response (e.g. increased peak ƒ_Hmax_) that likely influence the ability of fish to cope with natural acute heat challenges could be missed. These rapid, transient changes likely vary with the rate and magnitude of thermal variation, thus future studies that examine the extent to which such changes occur with more gradual, seasonal-like shifts in temperature would be of great benefit for conservation considerations.

## 5 Conclusion

The present study shows that rainbow trout can adjust their ƒ_Hmax_ and cardiac thermal sensitivity over a short timescale (24–72 h), which may help mitigate acute thermal challenges as part of a, larger, multi-system acclimation response. Together with past research from the molecular to whole-organism level (Ekström et al., 2016; Fangue et al., 2014; Sutcliffe et al., 2020), the rapidity of the changes observed here indicates that cardiac phenotypes are likely in near constant flux in thermally variable environments. Such rapid plasticity provides support for the potential value of incorporating realistic thermal variation into acclimation studies (Morash et al., 2020). Furthermore, such plasticity should be considered when anticipating the consequences of physiological phenotypes in the context of exposure to acute thermal variation. Future studies examining changes in autonomic cardiac control and *in vivo* cardiac function in more detail (e.g. routine and maximum stroke volume and cardiac output) over the biologically time frames identified here would help develop a more comprehensive picture of the time course for cardiac thermal acclimation.

## CRediT authorship contribution statement

**Matthew J.H. Gilbert:** Conceptualization, Methodology, Study design, Software, Validation, Formal analysis, Investigation, Resources, Data curation, Writing – original draft, Visualization, Supervision, Project administration, Funding acquisition. **Olivia A. Adams:** Validation, Investigation, Data curation, Writing – review & editing. **Anthony P. Farrell:** Conceptualization, Methodology, Study design, Resources, Writing – review & editing, Supervision, Project administration, Funding acquisition.

## Declaration of competing interest

The authors declare that they have no known competing financial interests or personal relationships that could have appeared to influence the work reported in this paper.
